# Resveratrol—Potential Antibacterial Agent against Foodborne Pathogens

**DOI:** 10.3389/fphar.2018.00102

**Published:** 2018-02-19

**Authors:** Dexter S. L. Ma, Loh Teng-Hern Tan, Kok-Gan Chan, Wei Hsum Yap, Priyia Pusparajah, Lay-Hong Chuah, Long Chiau Ming, Tahir Mehmood Khan, Learn-Han Lee, Bey-Hing Goh

**Affiliations:** ^1^Biofunctional Molecule Exploratory Research Group, School of Pharmacy, Monash University Malaysia, Bandar Sunway, Malaysia; ^2^Novel Bacteria and Drug Discovery Research Group, School of Pharmacy, Monash University Malaysia, Bandar Sunway, Malaysia; ^3^Biomedical Research Laboratory, Jeffrey Cheah School of Medicine and Health Sciences, Monash University Malaysia, Subang Jaya, Malaysia; ^4^International Genome Centre, Jiangsu University, Zhenjiang, China; ^5^Division of Genetics and Molecular Biology, Faculty of Science, Institute of Biological Sciences, University of Malaya, Kuala Lumpur, Malaysia; ^6^School of Biosciences, Taylor's University, Subang Jaya, Malaysia; ^7^Advanced Engineering Platform, Monash University Malaysia, Subang Jaya, Malaysia; ^8^Division of Pharmacy, School of Medicine, University of Tasmania, Hobart, Australia; ^9^School of Pharmacy, KPJ Healthcare University College, Nilai, Malaysia; ^10^Asian Centre for Evidence Synthesis in Population, Implementation and Clinical Outcomes, Health and Well-Being Cluster, Global Asia in the 21st Century Platform, Monash University Malaysia, Subang Jaya, Malaysia; ^11^The Institute of Pharmaceutical Sciences, University of Veterinary and Animal Sciences, Lahore, Pakistan; ^12^Center of Health Outcomes Research and Therapeutic Safety, School of Pharmaceutical Sciences, University of Phayao, Phayao, Thailand

**Keywords:** resveratrol, antibacterial, foodborne, pathogens, antibiofilm

## Abstract

Bacterial foodborne pathogens are a significant health burden and the recent emergence of pathogenic resistant strains due to the excessive use of antibiotics makes it more difficult to effectively treat infections as a result of contaminated food. Awareness of this impending health crisis has spurred the search for alternative antimicrobials with natural plant antimicrobials being among the more promising candidates as these substances have good acceptability and likely low toxicity levels as they have long been used in traditional medicines. Resveratrol (3,5,4′-trihydroxystilbene) is a naturally occurring stilbenoid which has been gaining considerable attention in medical field due to its diverse biological activities - it has been reported to exhibit antioxidant, cardioprotective, anti-diabetic, anticancer, and antiaging properties. Given that resveratrol is phytoalexin, with increased synthesis in response to infection by phytopathogens, there has been interest in exploring its antimicrobial activity. This review aims to provide an overview of the published data on the antibacterial activity of resveratrol against foodborne pathogens, its mechanisms of action as well as its possible applications in food packing and processing; in addition we also summarize the current data on its potential synergism with known antibacterials and future research and applications.

## Introduction

Foodborne illnesses resulting from of ingestion of food or drinks contaminated with pathogenic microorganisms represent a global health concern (Lv et al., 2011). Transmission of foodborne pathogens from contaminated food is a common health concern, the causative agents from bacteria, viruses, parasites, chemicals and prions. Kirk et al. (2015) estimated there were 2 billion cases of foodborne diseases in 2010 alone and over 1 million deaths with 78.7 million Disability Adjusted Life Years (DALYs) reported. About 582 million foodborne diseases cases were due to transmission from contaminated food, and the leading causative agent of foodborne illness was Norovirus which caused 125 million cases whereas *Campylobacter* spp. caused 96 million illness. The highest burden was diarrhea due to infection by non-typhoidal *Salmonella enterica* (NTS) which resulted in 4.07 million DALYs (Kirk et al., 2015).

The continuous use of antimicrobial agents like antibiotics is one of the main reasons antibiotic resistant bacteria strains have developed. Overuse of antibiotics in agriculture where they are used not only for treatment of infections but also for promoting animal growth is a significant public health risk as it promotes the of emergence and spread of antibiotic resistant bacteria from food producing animals (Tan et al., 2014). For example, the use of beta-lactams and ciprofloxacin resulted in the emergence of resistant strains of *Campylobacter* spp. and the use of streptomycin, sulfisoxazole and tetracycline resulted in development of resistance in *Escherichia coli* (Das et al., 2017). Since the introduction of tetracycline in 1957, the proportion of tetracycline resistant *E. coli* isolated from poultry increased from 3.5 to 63.2% in United Kingdom from 1957 to 1961 (Tellez et al., 2013). In short, the rise in antibiotic resistance has led to a decrease in the effectiveness of many antibiotics—a scenario which has tremendous implications for the pharmaceutical, medical, and food industries.

Given the significant morbidity and mortality associated with foodborne pathogens, the pharmaceutical industry is investing heavily in research looking for new inhibitory compounds from natural sources that can be developed into new anti-microbial drugs. Plant based medications have deep roots in medicinal lore, with mankind having relied on natural products derived from plants, animals, and microbes as remedies to treat illnesses for a very long time (Tan et al., 2015, 2017; Tang et al., 2016; Chan et al., 2017; Ser et al., 2017). In fact, in current clinical medicine, many modern drugs actually originated from plants which are rich in chemically diverse compounds and scaffolds (Fabricant and Farnsworth, 2001; Ser et al., 2016; Yong et al., 2017). In recent years, there has been increased interest in research seeking plant derived antimicrobials as an alternative to antibiotics. In addition to their efficacy in terms of antimicrobial properties, a significant advantage of these substances is that bacteria are less likely to develop resistance against them as they are acting on more than one target in cells (Burt, 2004). Also, certain plant derived antimicrobials are labeled as Generally Recognized as Safe (GRAS), giving them a “greener” image that is more acceptable to consumers (Xi et al., 2011). All these factors in combination suggest that natural antimicrobials have significant healthcare and market value given they match the increase in demand of greener additives by the consumers, supporting the value of research of plant based antimicrobials as potential biopharmaceutical products (Lai et al., 2017).

**Figure 1:**
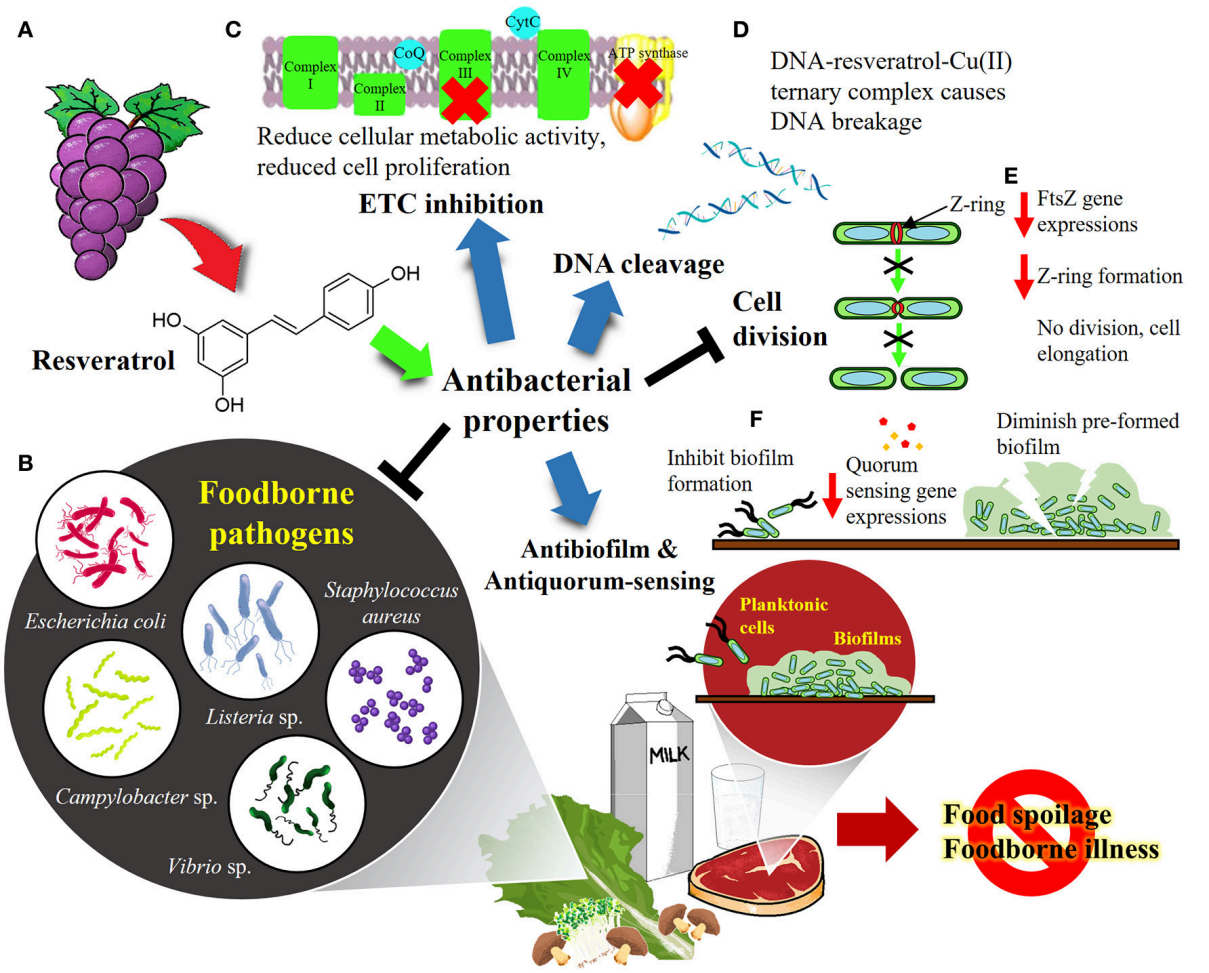
The overview of resveratrol as antibacterial agent against foodborne pathogens. **(A)** Resveratrol is found abundantly in the skin and leaves of grapevine (*Vitis vinifera*). **(B)** Resveratrol has been shown to exhibit promising antibacterial properties against variety of foodborne pathogens such as *E. coli, Listeria* sp., *Staphylococcus aureus, Campylobacter* sp. and *Vibrio* sp. **(C)** Resveratrol was shown to inhibit electron transport chain (ETC) and F0F1-ATPase, leading to reduced cellular energy production and thus proliferation of the microorganisms. **(D)** Resveratrol binds to Cu(II) to form a Cu(II)-peroxide complex, which binds DNA to form DNA-resveratrol-Cu(II) ternary complex, leading to DNA scission and breakage. **(E)** Resveratrol inhibits cell division by downregulating FtsZ gene which responsible for the formation of Z-ring required for prokaryotic cell division. **(F)** Resveratrol was demonstrated to be an effective antibiofilm and antiquorum sensing agent that prevents biofilm formation as well as disrupts the pre-formed biofilm. Overall, resveratrol could be developed into biopharmaceutical product as well as food preservatives in food industry to cope with food spoilage and foodborne outbreaks.

Among the various groups of plant based natural products, resveratrol (3,5,4′-trihydroxystilbene) seems highly promising for its therapeutic applications. It is a compound present in grape skins and wine, and is known to exhibit multi-spectrum therapeutic applications having been reported to exhibit anticancer, antiangiogenic, immunomodulatory, cardioprotective, and antioxidant activity. Although there is still very limited work on the antibacterial activity of resveratrol, it has been shown that resveratrol exhibits antibacterial activity against several Gram-positive and Gram-negative foodborne bacteria (Chan, 2002; Tegos et al., 2002; Paulo et al., 2010; Paolillo et al., 2011; Alvarez et al., 2012, 2013; Kumar et al., 2012b; Plumed-Ferrer et al., 2013; Augustine et al., 2014; Ferreira et al., 2014; Moran et al., 2014; Promgool et al., 2014; Subramanian et al., 2014; Duarte et al., 2015; Kim et al., 2015; Makwana et al., 2015; Ferreira and Domingues, 2016; Liu et al., 2016; Seukep et al., 2016; Silva et al., 2016; Surendran Nair et al., 2016; Klancnik et al., 2017; Lai et al., 2017; Lee and Lee, 2017; Oliveira et al., 2017). This review aims to provide an overview of the published data on the antibacterial activity of resveratrol against foodborne pathogens, its mechanisms of action as well as its possible applications in food packing and processing; in addition we also summarize the current data on its potential synergism with known antibacterials and future research and applications. Figure 1 depicts the overview of this review.

## Overview and rationale of resveratrol as antimicrobial agent

**Figure 2:**
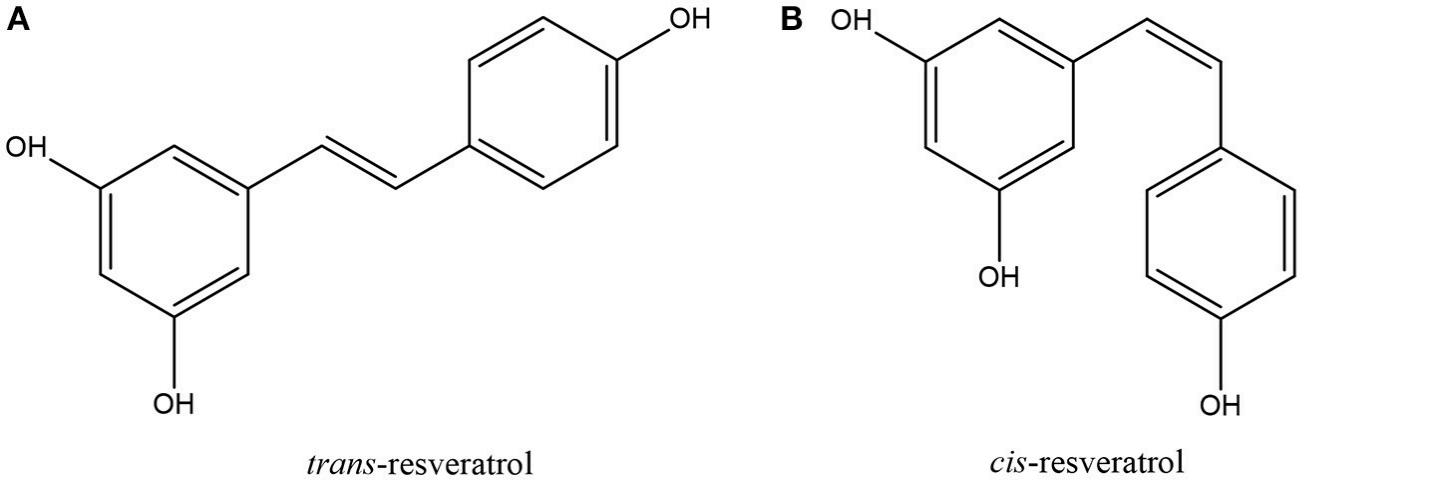
Chemical structures of **(A)** *trans-*resveratrol and **(B)** *cis*-resveratrol.

Resveratrol is a naturally occurring phytoalexin belonging to the stilbene family of phenolic compounds; it exists in either *trans* or *cis* geometrical isomers with the *trans* isomer (Figure 2) being the predominant isomer in plants (Filip et al., 2003; Catalgol et al., 2012) and hence forming the focus of this review. First isolated by Takaoka in 1940 from the roots of white hellebore (*Veratrum grandiflorum O. Loes*), the compound was later named resveratrol since it is a resorcinol derivative from *Veratrum* species (Catalgol et al., 2012). As a polyphenolic compound, resveratrol has been reported in over 100 medical and edible plants such as *Polygonum cuspidatum, Arachis hypogaea, Yucca Shidigera, Cassia quinquangulata, Rheum rhamponticum*, and many more (Rocha-Gonzalez et al., 2008; Chachay et al., 2011). In 1992, resveratrol, as the active component in red wine, was believed to be responsible for the phenomenon known as the “French paradox” whereby epidemiological data shows that the French population have a lower mortality rate from cardiovascular diseases (CHD) despite a relatively high dietary fat consumption (Catalgol et al., 2012; Tome-Carneiro et al., 2013). Renaud and de Lorgeril (1992) proposed that the lower mortality rate of the French population—in spite of their relatively fatty diet—from CHD was due to the moderate consumption of resveratrol-containing wine, and suggested that the consumption of wine inhibited platelet aggregation thus leading to reduced risk of CHD (Renaud and de Lorgeril, 1992). In the following years, resveratrol was very much in the spotlight with the first evidence published of it potential to prevent carcinogenesis in mice (Jang et al., 1997). Since then, resveratrol has received considerable attention for its health promoting properties such as its antioxidant, cardioprotective, anti-diabetic, anticancer, and antiaging properties (Jung et al., 2009; Fernández-Mar et al., 2012).

Phytoalexins are naturally-occurring, low molecular weight secondary metabolites produced by plants in respond to adverse conditions such as biotic stress from bacteria, fungi, viruses, and abiotic stress from chemical treatment (Filip et al., 2003; Pangeni et al., 2014). For instance, resveratrol is known as a major stilbene phytoalexin which is produced as an active defense mechanism to protect grape plants from phytopathogens. During *Botrytis cinerea* fungal infection, the amount of resveratrol was found to increase in the skin of infected grapes (Jeandet et al., 1995). Moreover, the synthesis of resveratrol was also increased in grapevine berries during fungal infection by *Aspergillus carbonarius* (Bavaresco et al., 2008). Similarly, the bacterium belonging to genus *Bacillus* was also shown to stimulate the production of resveratrol in the leaves of grapevine plants (Paul et al., 1998). Besides that, the grapevine *Vitis vinifera* which is rich in resveratrol has also been used as popular folk remedy to treat diarrhea, hemorrhage, varicose veins, inflammatory disorder and also heal wounds and drain furuncles (Bombardelli and Morazzoni, 1995; Pari and Suresh, 2008; Orhan et al., 2009). Resveratrol, as a logical target, has been investigated in the same manner as other plant derived phenolic compounds (Chan et al., 2016), in many previous studies as alternatives to antibiotics (Burt, 2004). The work so far appears encouraging with resveratrol having been reported to exhibit antimicrobial properties against bacteria (Hwang and Lim, 2015), yeasts (Jung et al., 2007) and fungi (Jung et al., 2005).

## *In vitro* antibacterial tests of resveratrol

Several analytical methods have been employed to evaluate the antibacterial activity of resveratrol against foodborne bacterial pathogens; with the two most important methods used being the disk diffusion assay and broth microdilution assay which enable an estimate of the efficacy of resveratrol against a tested pathogen. Furthermore, several other advanced techniques have also been adopted to evaluate the antibacterial activity of resveratrol such as time kill analysis, scanning electron microscopy, crystal violet biofilm assay, and quorum sensing inhibition assay (Burt, 2004; O'toole, 2011). Based on the available literature, resveratrol was demonstrated to exhibit differential antibacterial activities against different strains of foodborne pathogens including *Staphylococcus aureus, Bacillus cereus, Bacillus subtilis, and Listeria monocytogenes, E. coli* O157:H7, *Salmonella* Typhimurium, *Vibrio cholera, Campylobacter jejuni, Campylobacter coli, Arcobacter butzleri*, and *Arcobacter cryaerophilus* (Tables 1, 2).

**Table 1:** MIC of resveratrol against Gram-negative bacteria.

**Gram types**	**Bacterial pathogens**	**Bacteria strains**	**Source of bacterial strain**	**MIC**	**MBC**	**References**
Gram-negative	*Escherichia coli*	BW25113	derivative of *E. coli* K-12 W1485	400 μg/mL	–	Liu et al., 2016
		BCRC10675	Isolated from urine	521 μg/mL	–	Lai et al., 2017
		ATCC 25922	clinical isolate	> 400 μg/mL	–	Paulo et al., 2010
		TISTR 780	Isolated from feces	128 μg/mL	–	Promgool et al., 2014
		ATCC 8739	Isolated from feces	64 μg/mL	64 μg/mL	Seukep et al., 2016
		AG100ATet	–	128 μg/mL	256 μg/mL	Seukep et al., 2016
		AG102	–	32 μg/mL	32 μg/mL	Seukep et al., 2016
		MTCC 2622	Isolated from stools of diphtheria convalescent patients	32 μg/mL	64 μg/mL	Kumar et al., 2012b
		K-12	–	500 μg/mL	–	Tegos et al., 2002
	*Escherichia coli O157:H7*	BCRC 15374	Isolated from human feces	521 μg/mL	–	Lai et al., 2017
	*Salmonella typhimurium*	ATCC 13311	Isolation from human feces (food poisoning)	> 400 μg/mL	–	Paulo et al., 2010
		Unidentified	–	5 μg/mL	–	Lee and Lee, 2017
		TISTR 292	Isolated from feces of man with food poisoning	128 μg/mL	–	Promgool et al., 2014
		ST329	–	500 μg/mL	–	Tegos et al., 2002
	*Vibrio cholera* O1	MCVO9	Clinical isolate from cholera outbreak	60 μg/mL	–	Augustine et al., 2014
		ATCC 39315	Isolated from feces of cholera patient	0.625 μg/mL	–	Kim et al., 2015
	*Campylobacter coli*	873	Isolate from feces of patient with acute gastroenteritis	50 μg/mL	–	Duarte et al., 2015
	*Campylobacter jejuni*	225421	Isolated from fresh poultry meat	100 μg/mL	–	Duarte et al., 2015
		K49/4	Isolated from food	313 μg/mL	–	Klancnik et al., 2017
		NCTC 11168	Isolated from human feces (reference strain)	313 μg/mL	–	Klancnik et al., 2017
	*Arcobacter butzleri*	AB36/11	Isolated from poultry caecum	100 μg/mL	–	Duarte et al., 2015
		INSA 776	Isolated from feces of patient with diarrhea and abdominal pain	100 μg/mL	–	Duarte et al., 2015
		LMG 10828	Isolated from feces from man with diarrhea	100 μg/mL	–	Ferreira et al., 2014
	*Arcobacter cryaerophilus*	LMG 10829	Isolated from human blood	500 μg/mL	–	Ferreira et al., 2014

**Table 2:** MIC of resveratrol against Gram-positive bacteria.

**Gram types**	**Bacterial pathogens**	**Bacteria strains**	**Source of bacterial strain**	**MIC**	**MBC**	**References**
Gram-positive	*Staphylococcus aureus*	RN450	–	150 μg/mL	–	Liu et al., 2016
		ATCC 25923	Clinical isolate	100 μg/mL	–	Paulo et al., 2010
		ATCC 25923	Clinical isolate	200 μg/mL	> 400 μg/mL	Oliveira et al., 2017
		MTCC 902	Isolated from man with septic arthritis	32 μg/mL	32 μg/mL	Kumar et al., 2012b
		8325-4	–	125 μg/mL	–	Tegos et al., 2002
		BCRC12655	Isolated from sliced turkey	130–260 μg/mL		Lai et al., 2017
		BCRC10780	Isolated from human pleural fluid	130–260 μg/mL	–	Lai et al., 2017
	*MRSA*	COL	Clinical isolate	350 μg/mL	> 800 μg/mL	Qin et al., 2014
	*Bacillus cereus*	ATCC 11778	–	50 μg/mL	–	Paulo et al., 2010
		TISTR 687	–	64 μg/mL	–	Promgool et al., 2014
	*Bacillus subtilis*	MTCC 2756	Isolated from Kalimpong, India	16.5 μg/mL	32 μg/mL	Kumar et al., 2012b
	*Listeria monocytogenes*	LMG 16779 serovar 1/2a	–	200 μg/mL	–	Ferreira and Domingues, 2016
		LMG 16780 serovar 1/2b	–	200 μg/mL	–	Ferreira and Domingues, 2016
		LMG 13305 serovar 4b	–	200 μg/mL	–	Ferreira and Domingues, 2016
	*Listeria innocua*	LOP 9	Isolated from sheep milk	200 μg/mL	–	Ferreira and Domingues, 2016
		LMG 16779	–	200 μg/mL	> 400 μg/mL	Oliveira et al., 2017

According to the data from previous studies, the minimum inhibitory concentration (MIC) of resveratrol against Gram-negative ranged from 0.625–521 μg/mL while the MIC of resveratrol against Gram-positive bacteria ranged from 16.5–260 μg/mL. In general, previous studies indicated that resveratrol exhibits better activity against Gram-positive bacteria than Gram-negative bacteria. Using a simple analysis based on the data collected, it was found that the mean MIC of resveratrol against Gram-positive bacteria was 141.83 ± 18.30 μg/ml while against Gram-negative bacteria was 224.82 ± 39.41 μg/ml. For instance, Paulo et al. (2010) reported that the MIC of resveratrol toward all tested Gram-negative bacteria were more than 400 μg/mL while MIC toward Gram-positive bacteria ranged from 50 to 100 μg/mL. They proposed that the difference in susceptibilities toward resveratrol might be attributed to the presence of a hydrophilic outer membrane in Gram-negative bacteria which is absent in Gram-positive bacteria. The hydrophilic outer membrane may act as a protective layer resisting the diffusion of hydrophobic molecules like resveratrol from penetrating the bacterial cell, thereby reducing the efficacy of the antibacterial action of resveratrol (Paulo et al., 2010). Besides that, the presence of degradative and detoxifying enzymes in the periplasmic space of Gram-negative bacteria could be another contributing factor to reduced activity of resveratrol due to breakdown of the resveratrol molecule by these enzymes (Beveridge, 1999). Another possibility of the observed lower sensitivity demonstrated by Gram-negative bacteria toward resveratrol could be the presence of multidrug resistance pumps (MDRPs) on their cell surface which may be able to extrude resveratrol from the cell. This appears to be unique to Gram negative rather than Gram positive organisms as demonstrated by Tegos et al. (2002) who reported that the MIC of resveratrol for *S. aureus* and *E. coli* was 125 μg/mL and 500 μg/ml respectively. However, the MIC of resveratrol for *E. coli* decreased by 16-fold in the presence of a MDRPs inhibitor while for *S. aureus*, the MIC only decreased by 2-fold which suggested the effect of MDRPs in reducing the activity of resveratrol in Gram-negative bacteria (Tegos et al., 2002). Nevertheless, it is still difficult to draw any definite conclusions about the difference in susceptibility between Gram-positive and Gram-negative bacteria toward resveratrol. A study by Taguri et al. (2006) demonstrated that the susceptibility to phenolic compounds was dependent on the bacterial species with Gram-staining not necessarily correlating with the antimicrobial efficacy.

Combinatorial therapy may be one strategy to address the differential susceptibility toward resveratrol and development of resistance, as it could potentially enhance the efficacy of antimicrobials as well as to prevent emergence of resistant strains by exploiting the synergism between multiple antimicrobials. Kumar et al. (2012a) reported the existence of synergistic activity of resveratrol and antibiotics, showing improved antibacterial activity compared to treatment of resveratrol or the antibiotic alone. The study revealed the synergism between resveratrol and ciprofloxacin while additive effect between resveratrol and cefotaxime against both Gram-negative or Gram-positive bacteria (Kumar et al., 2012a). It may be pertinent for future research to investigate the interactions between resveratrol with other commonly used antibiotics in clinical medicine in order to fully exploit resveratrol as a novel antibacterial agent.

## Possible mode of actions of resveratrol against foodborne bacteria

Understanding the mechanisms underlying the actions of a potential antibacterial molecule is key to assisting in identifying the likely targets and predicting the possible outcomes of resistance mechanisms that target bacteria may develop toward the molecule. The exact mechanism of the antibacterial activities of resveratrol against bacterial pathogens are still elusive. Nevertheless, several studies have reported possible antibacterial mechanism of action of resveratrol which include DNA damage (Subramanian et al., 2016), cell division impairment (Haranahalli et al., 2016), oxidative membrane damage (Subramanian et al., 2014), and also metabolic enzymes inhibition (Dadi et al., 2009).

Many antibiotics induce oxidative stress in target bacteria by producing ROS that cause oxidative damage to DNA, subsequently leading to cell death (Goswami et al., 2006). Even though the antioxidant properties of resveratrol are attributed to it being a free radical scavenger and its ability to promote activity of various antioxidant enzymes, depending on the concentration of resveratrol and the target cell types, resveratrol can also exhibit pro-oxidant properties (de la Lastra and Villegas, 2007). In fact, recent studies indicated that resveratrol did not result in generation of ROS in *E. coli* (Subramanian et al., 2014; Haranahalli et al., 2016)*;* the study instead suggested that the inhibition of *E. coli* by resveratrol may be mediated through site-specific oxidative membrane damage (Subramanian et al., 2014) though the mechanism behind the membrane damage is still under investigation.

**Figure 3:**
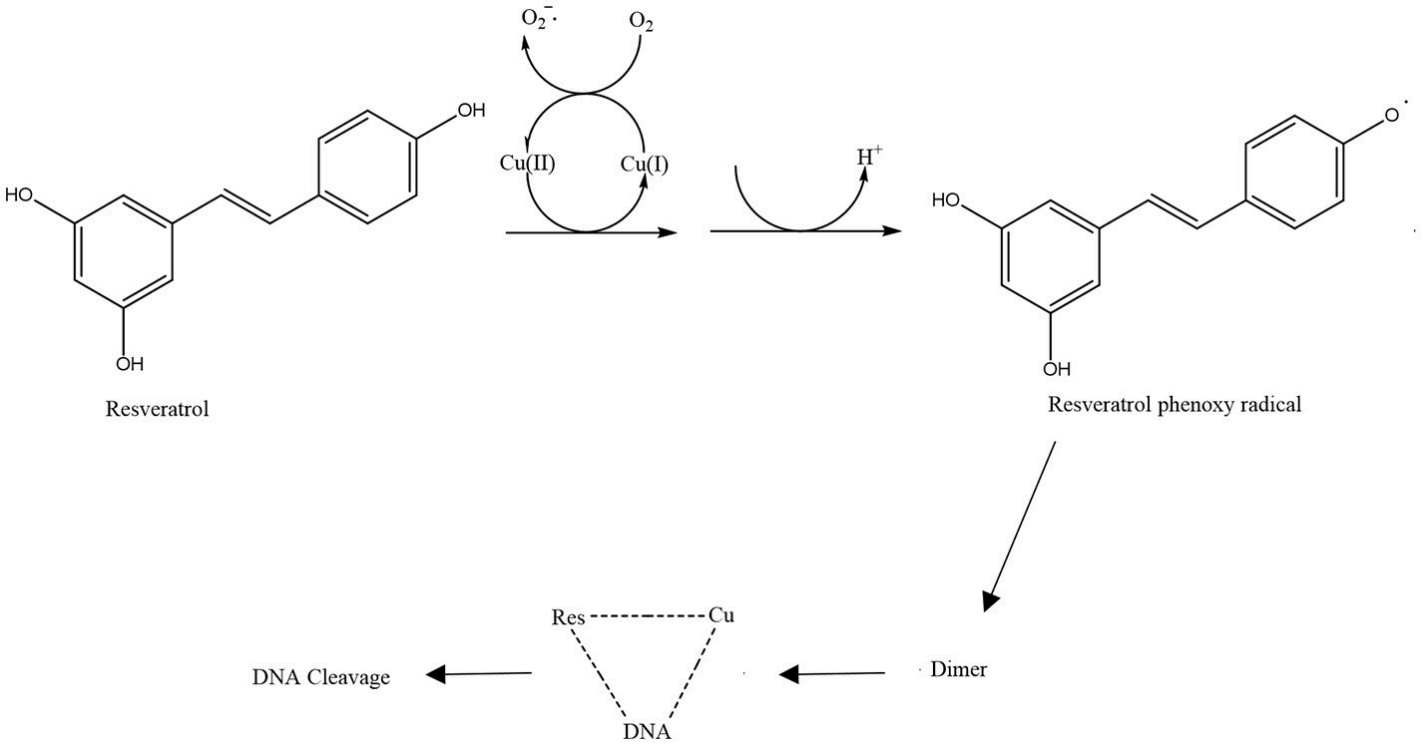
Schematic diagram of resveratrol pro-oxidant activity (de la Lastra and Villegas, 2007).

**Figure 4:**
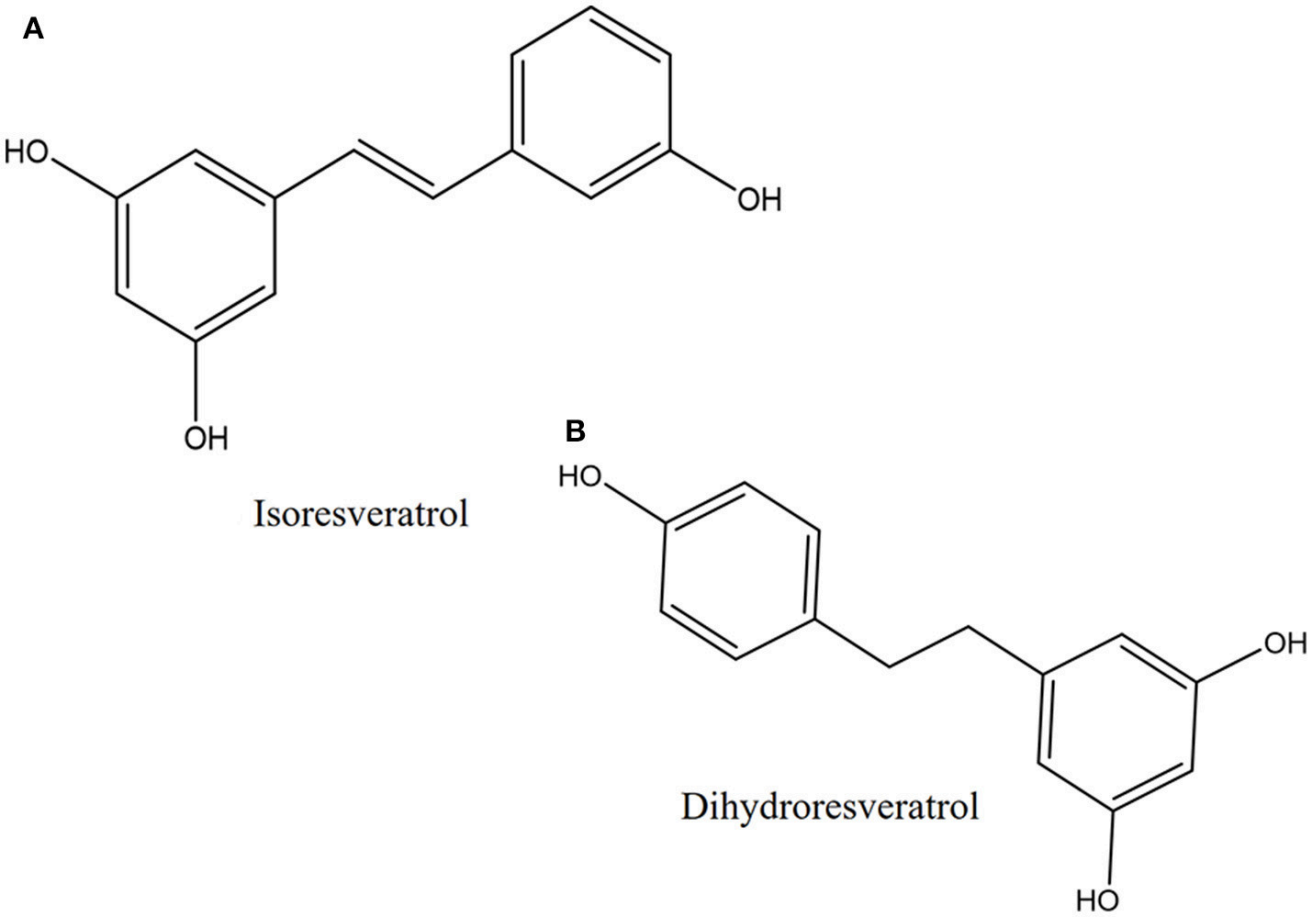
Chemical structures of **(A)** isoresveratrol and **(B)** dihydroresveratrol.

Another proposed antibacterial mechanism of resveratrol may be through the generation of Cu(II)-peroxide complex upon reduction of Cu(II) to Cu(I) with resultant DNA cleavage and cell death (Fukuhara and Miyata, 1998). Fukuhara and Miyata (Fukuhara and Miyata, 1998) proposed that resveratrol binds to Cu(II) to form a Cu(II)-peroxide complex, which leads to reduction of Cu(II) to Cu(I). The Cu(II)-peroxide complex binds DNA to form DNA-resveratrol-Cu(II) ternary complex and this leads to DNA scission and breakage (Figure 3). The reduction of Cu(II) to Cu(I) is necessary for DNA cleavage to occur. When buthacuprione, a Cu(I) chelating agent, was added to solution containing resveratrol and Cu(II), there was a reduction in the levels of the nicked form of DNA produced from supercoiled DNA, indicating reduction in DNA cleavage (Fukuhara and Miyata, 1998). Additionally, the number and positions of the hydroxyl group in the stilbene backbone also have an effect on DNA cleavage activity. Resveratrol with a 4-hydroxy group has pro-oxidant activity but isoresveratrol (Figure 4A), an analog of resveratrol with a 3-hydroxy group did not reduce Cu(II) and cause DNA cleavage. The 4-hydroxy group is responsible for its high affinity binding to both Cu(II) and DNA in forming a ternary complex to cleave DNA efficiently. Furthermore, dihydroresveratrol (Figure 4B) was shown to have decreased DNA cleaving ability compared to resveratrol, which indicates the importance of the double bond in resveratrol which not only provides planarity to the structure for effective DNA binding, but also confers the stability of the 4-oxy radical form (Fukuhara et al., 2006). A more recent study by Subramanian et al. (2016) suggested that DNA damage induced by resveratrol in *E. coli* could be a much later event occurring only after the membrane integrity has been compromised, indicating that there is much to be explored in order to fully understand the mechanism of reservatrol's action.

Another proposed mechanism of resveratrol's antibacterial activity is through the inhibition of the electron transport chain (ETC) and F0F1-ATPase, thereby inhibiting the proliferation of pathogenic microorganisms by reducing cellular energy production (Madrigal-Perez and Ramos-Gomez, 2016). A study by Dadi et al. (2009) found that resveratrol inhibited *E. coli* ATP synthase activity, leaving only 60% residual activity. Resveratrol (at 100 μM) was also found to decrease complex III activity by competing with coenzyme Q, leading to about 20% reduction in enzyme activity (Zini et al., 1999). In another study, resveratrol was found to inhibit enzymatic activity of F0F1-ATPase/ATP synthase in rat brain and liver in a concentration-dependent manner (Zheng and Ramirez, 2000). Similarly, a significant reduction of metabolic activity was observed in the genus *Arcobacter* bacteria upon treatment with resveratrol (Ferreira et al., 2014).

Reservatrol also appears to have an inhibitory action on cell division through its actionon filamentous temperature sensitive protein (FtsZ), a GTP-dependent prokaryotic cell division protein responsible for cell division through formation of dynamic Z-ring in the middle of cell. Inhibiting expression of FtsZ gene leads to a decrease in Z-ring formation in cells and therefore inhibition of cell division (Haranahalli et al., 2016). By using phase contrast microscopy, Hwang and Lim (2015) observed that the length of resveratrol treated *E. coli* cells increased in a dose-dependent manner, suggesting that the elongation was due to interference of septum formation, which therefore indicated a failure of cell division. To confirm the effect of resveratrol on Z-ring formation, they used an *E. coli* strain JW0093 containing plasmid that expressed one or more Z-rings. Confocal microscopy showed that *E. coli* expressed Z-ring formation in the absence of resveratrol while treatment with resveratrol diminished the appearance of Z-rings. Western blot analysis was used to further confirm the inhibition of FtsZ expression by resveratrol, the analysis of which showed a decrease in FtsZ levels in *E. coli* treated with resveratrol. These results led to them conclude that the antibacterial activity of resveratrol was attributed to suppression of FtsZ gene expression, thereby inhibiting Z-ring formation and cell division (Hwang and Lim, 2015).

## Antibiofilm and antivirulence activities of resveratrol against foodborne bacteria

One of bacteria's survival strategies is the ability to form biofilms where they adhere to various surfaces and form sessile biofilm communities using a self-produced extracellular polymeric matrix. These biofilms produced by pathogenic foodborne bacteria can cause serious health risks due to their inherent resistance to antimicrobial agents, host defenses and external stresses, which make them difficult to eradicate and can be an additional factor leading to antibiotic resistance (Cho et al., 2013, 2015; Lee J. H. et al., 2014). In food industries, the biofilms of spoilage or pathogenic bacteria represent a considerable problem as they form on food surfaces as well as the processing environment, leading to postprocessing contamination and cross-contamination. As an intercellular communication mechanism, quorum sensing regulates a variety of physiological functions of bacteria including the virulence and biofilm formation (Deep et al., 2011) which could limit bacterial proliferation in foods and food spoilage. Hence, new alternatives to antibiotics that could eradicate bacterial biofilms and diminish virulence of foodborne bacteria could be a promising strategy to ensure food safety and quality (Cho et al., 2013).

Considering the complexity and heterogeneity of biofilm structure, several parameters have to be taken into account when selecting the proper measurement endpoint for antibiofilm screens (Fallarero et al., 2014), which includes the measurement of the biomass, biofilm viability as well as the extracellular matrix of the biofilm. The literature documents several methods that have been employed to assess the antibiofilm activity of resveratrol; these methods include the quantification of the biofilm using crystal-violet dye (Lee K. et al., 2014; Qin et al., 2014), quantification of bacterial viability using metabolic assays (MTT) (Duarte et al., 2015) and microscopic evaluation using scanning electron microscope and confocal laser scanning microscope (Lee J. H. et al., 2014; Cho et al., 2015). Furthermore, studies also further investigated the antibiofilm associated mechanisms of resveratrol by assessing the effect of resveratrol on the genes associated to the regulation of biofilm development (Qin et al., 2014). Several studies have reported the biofilm inhibitory activity of resveratrol against both Gram positive and Gram-negative foodborne pathogens (Tables 3, 4). Differential activities of resveratrol in inhibiting biofilm formation and eradicating biofilm already formed were observed against specific species of foodborne pathogens. Previous work demonstrated that resveratrol appears effective in inhibiting biofilm formation by *S. aureus* (Moran et al., 2014; Qin et al., 2014), *L. monocytogenes* (Ferreira and Domingues, 2016), *E. coli* O157:H7 (Lee et al., 2013), *Campylobacter* sp. (Duarte et al., 2015), and *V. cholerae* (Augustine et al., 2014).

**Table 3:** Antibiofilm activity of resveratrol against Gram-positive bacteria.

	**Bacterial pathogens**	**Bacteria strains**	**Source of bacterial strain**	**Applied concentration**	**Assay**	**Observations**	**References**
Gram- positive	Enteropathogenic *S. aureus* CECT 59	ATCC 9144	–	100 μg/mL	Crystal violet biofilm assay	Inhibition of *S. aureus* biofilm formation by between 20% and 45%. Antagonistic anti-biofilm effect present with combination of resveratrol and genistein or protocateuchic acid	Moran et al., 2014
	*S. aureus*	ATCC 6538	Isolated from human lesion	100 μg/mL	Crystal violet biofilm assay	No significant inhibitory effect on biofilm formation	Lee K. et al., 2014
		ATCC 6538	Isolated from human lesion	20–100 μg/mL	Crystal violet biofilm assay	No significant inhibitory effect on biofilm formation at concentrations up to 100 μg/mL	Cho et al., 2015 Qin et al., 2014
	*MRSA*	COL	Clinical isolate	100 μg/mL, 150 μg/mL	Crystal violet biofilm assay	39.85 % inhibition of MRSA biofilm at 100 μg/mL resveratrol. 23.42 % removal of preformed biofilm at 150 μg/mL resveratrol.	
					SEM	Thinner biofilm post-treatment with resveratrol	
	*Listeria monocytogenes*	LMG 16779 serovar 1/2a	–	20, 100, 200 μg/mL	Crystal violet biofilm assay	Similar % biofilm inhibition for LMG 16779, LMG 16780 and LOP9 at 100 μg/mL and 200 μg/mL resveratrol. LMG serovar 1/2a LMG 16679 (most commonly found serovar in food processing plant) is the most sensitive and LMG 13305 is the most resistant to biomass inhibition at lowest tested concentration (20 μg/mL).	Ferreira and Domingues, 2016
		LMG 16780 serovar 1/2b	–				
		LMG 13305 serovar 4b	–				
	*Listeria innocua*	LOP9	–				

**Table 4:** Antibiofilm activity of resveratrol against Gram-negative bacteria.

**Gram types**	**Bacterial pathogens**	**Bacteria strains**	**Source of bacterial strain**	**Applied concentration**	**Assay**	**Observations**	**References**
Gram-negative	Enterohemorrhagic *E. coli* O157:H7	ATCC 43895	Isolated from raw hamburger meat implicated in hemorrhagic colitis outbreak	10, 20, 50 μg/mL	Crystal violet biofilm assay	Reduction of EHEC biofilm production as low as 10 μg/mL	Lee et al., 2013
		EDL 933	Isolated from raw hamburger meat implicated in hemorrhagic colitis outbreak		Confocal laser microscopy (fluorescent)	Reduction of EHEC biofilm production on glass surface at 50 μg/mL	
	commensal *E. coli* K-12	BW 25113	–	50, 100 μg/mL	Crystal violet biofilm assay	No inhibition of formation of biofilm of all four commensal *E. coli*	Lee et al., 2013
		MG1655	Derived from parent strain W1485				
		TG1	–				
		DH5α	–				
	*Campylobacter coli*	873	Isolate from feces of patient with acute gastroenteritis	6.25–200 μg/mL	Crystal violet biofilm assay	200 μg/mL resveratrol resulted in 63 −94 % biofilm inhibition in all 4 strains of bacteria. For established biofilm, 200 μg/mL resveratrol resulted in 80 % biofilm inhibition for both Campylobacter strains and A.butzleri AB36/11 and a reduction of 64% for the A. butzleri INSA 776. For all strains, even at subinhibitory resveratrol concentrations, inhibited biofilm formation as well as diminished established biofilm.	Duarte et al., 2015
	*Campylobacter jejuni*	225421	Isolated from fresh poultry meat				
	*Arcobacter butzleri*	AB36/11	Isolated from poultry caecum				
		INSA 776	Isolated from feces of patient with diarrhea and abdominal pain				
	*Campylobacter jejuni*	K49/4	Isolated from food	12.5 – 1565 μg/mL		Subinhibitory concentration (12.5–200 μg/mL) resulted in 40 % inhibition of biofilm formation and 20–30% inhibition at other lower concentrations. At MIC, two times and five times MIC, even higher percentage biofilm inhibition was achieved	Klancnik et al., 2017
	*Vibrio cholerae O1*	MCVO9	Clinical isolate from cholera outbreak	10, 15, 20, 25, 30μg/mL	Crystal violet biofilm assay	Concentration dependent inhibition of % biofilm formation. 15 – 30 μg/mL resveratrol inhibits biofilm formation by 64–85%. At 10 μg/mL, inhibition was not significant.	Augustine et al., 2014
				15,20 μg/mL	Confocal laser microscopy	Reduction of cells adhered to coverslip. Significant difference in thickness of biofilm in treated and control. At 15 μg/mL and 20 μg/mL, the measured thickness of biofilm was 18 μm and 15 μm respectively. In untreated cells, the thickness measured was 70 μm.	
	*Vibrio cholerae*	ATCC 39315	Isolated from feces of cholera patient	10 μg/mL	Crystal violet biofilm assay	No significant reduction in % biofilm formation	Kim et al., 2015

Qin et al. (2014) demonstrated that resveratrol at 100 μg/mL caused 39.85% inhibition of methicillin-resistant *S. aureus* (MRSA) biofilm formation and at 150 μg/mL, caused 23.42% removal of preformed biofilm. This was also visualized by SEM where 100 μg/mL and 150 μg/mL resveratrol both resulted in thinner biofilm post-treatment. In addition, the combination of resveratrol 150 μg/mL and vancomycin 8 μg/mL resulted in 55.43% removal of preformed biofilm which suggested potential of resveratrol as adjunct therapy in MRSA biofilm infection for its synergistic activity (Qin et al., 2014). It was proposed the antibiofilm activity of resveratrol might be due to its ability to cause interference in quorum sensing thus affecting synthesis of surface proteins and capsular polysaccharides—downregulation of genes (*cap*5ABCFG) responsible for capsular polysaccharide synthesis was reported with resveratrol treatment at 100 μg/mL. The combination of resveratrol and vancomycin was also found to downregulate the virulence gene *clfA* and biofilm gene *spoVG*. The authors proposed that the synergistic activity of the resveratrol/vancomycin combination was due to vancomycin induced upregulation of the genes associated with purine metabolism (*pur*ADHN) which was observed in the combination treatment but not in resveratrol alone (Qin et al., 2014). As such, the combinatorial treatment was more effective in removing preformed biofilm as it induced purine metabolism and reduced capsular polysaccharide synthesis (Qin et al., 2014). Furthermore, there was additional evidence showing the inhibitory effect of resveratrol on biofilm formation by enteropathogenic *S. aureus* in which resveratrol at sub-inhibitory concentrations of 100 μg/mL exerted 20 to 45% antibiofilm activity (Moran et al., 2014).

However, there have been also been several previous studies reporting negative inhibitory effect of resveratrol against biofilm formation by methicillin-sensitive *S. aureus* (MSSA) (Lee K. et al., 2014; Cho et al., 2015). Lee K. et al. (2014) reported *trans*-resveratrol at 100 μg/mL had no significant effect in reducing biofilm formation in *S. aureus* ATCC6538 when compared to control. Similarly, Cho et al. (2015) also reported *trans*-resveratrol had no antibiofilm activity on *S. aureus* ATCC6538 at concentrations up to 100 μg/mL. This data seems to highlight that the antibiofilm activity of resveratrol could be strain specific, particularly toward MRSA and enteropathogenic *S. aureus* but not *S. aureus* ATCC6538. The differential antibiofilm activity of resveratrol toward the different strains of *S. aureus* could be explained by the difference in the involvement of multiple molecules and factors including the adhesion factors, quorum sensing and capsular polymers possessed by the bacterial strains to form biofilm (Karatan and Watnick, 2009; Moran et al., 2014). Besides that, the divergent findings could also be related to the varying methodologies employed by respective studies in assessing the antibiofilm properties of resveratrol. Particularly, the spectroscopic assay using the crystal violet stain to quantify the total biofilm biomass is subject to several limitations, including species dependence, requirement for individual optimization of protocols (e.g., period for biofilm formation, development, maturation, staining) and it is also unable to discriminate between the different components in the biofilm (e.g., living and non-living cells; Pantanella et al., 2013; Bueno, 2014). To differentiate living or dead microorganisms within the biofilm, the crystal violet method could be coupled with bacterial viability assays such as conventional viable plate counting and metabolic assays such as MTT (Fallarero et al., 2014).

Contamination of food by *L. monocytogenes* is common in food processing environments due to their hardy growth characteristics and ability to attach to stainless steel and many surfaces to form biofilm (Donnelly, 2001). Often, *L. monocytogenes* is associated with foodborne disease outbreaks characterized by widespread distribution and high mortality rates. Thus, effective strategies to remove or inhibit biofilm formation by *L. monocytogenes* would be of tremendous value to the food processing industry. In 2016, Ferreira and Domingues (2016) demonstrated that resveratrol at MIC of 200 μg/mL produced approximately 60 to 70% biofilm inhibition against three *L. monocytogenes* strains (LMG 16779 serovar 1/2a, LMG 16780 serovar 1/2b, LMG 13305 serovar 4b) and *Listeria innocua* LOP 9 isolated from sheep's milk. They also reported that *L. monocytogenes* serovar 1/2a, the most commonly found serovar in food processing plants was the most sensitive strain toward biofilm inhibition by resveratrol (Ferreira and Domingues, 2016). This study claimed to be the first to demonstrate the inhibitory effect of resveratrol against biofilm formation by *L. monocytogenes* (Ferreira and Domingues, 2016).

*Trans*-resveratrol was also shown to be an effective antibiofilm and antiquorum sensing agent against enterohemorrhagic *E. coli* O157:H7 (EHEC) (Lee et al., 2013). Using *trans*-resveratrol at 10 μg/mL, biofilm formation as well as swimming and swarming abilities of EHEC were inhibited through down-regulation of several quorum sensing genes. They showed that resveratrol at 20 μg/mL downregulated the expression of curli genes (*csgA* and *csgB*), motility gene (*flhD, fimA, fimH*, and *motB*) and autoinducer-2 (AI-2) quorum sensing gene (*lsrA, luxS*, and *luxR*) (Lee et al., 2013). The study also demonstrated specific and selective inhibitory effect of *trans*-resveratrol against EHEC but did not harm the commensal *E. coli* strains, suggesting that possible application of resveratrol as an antibiofilm compound in combination with lower dose of antibiotics to maximize removal of pathogen without rendering development of antibiotic resistance while protecting commensal microflora (Lee et al., 2013).

*C. jejuni* and *C. coli* are the leading agents responsible for bacterial gastroenteritis in humans worldwide from consumption of contaminated poultry meat and products. They also form biofilms that are resistant to disinfectant and represent a major issue within the food industry. Several studies have been conducted to investigate the potential of resveratrol as an antibiofilm and antiquorum sensing agent against *Campylobacter* bacteria. Duarte et al. (2015) reported resveratrol at 200 μg/mL resulted in 63–93% biofilm inhibition in four tested bacteria strains (*C. coli* 873, *C. jejuni* 225421, and *A. butzleri* AB36/11 and INSA 776). With reference to preformed biofilm, 200 nμg/mL resveratrol caused 80% reduction in biofilm formation for both *Campylobacter* strains and *A. butzleri* AM36/11, and 64% reduction for *A. butzleri* INSA 776. The reported concentration-dependent antibiofilm activity of resveratrol in inhibiting biofilm formation and in diminishing preformed biofilm in all four bacteria strains even at sub-inhibitory concentration of 6.25–50 μg/mL suggests that there could be significant applications for this compound. The authors proposed that the effect of resveratrol on biofilm formation is related to interference in the expression of biofilm-related genes as shown in study by Lee et al. (2013), and the antibiofilm activity on preformed biofilm was related to the effect on quorum sensing (Reeser et al., 2007; Gölz et al., 2012). Klancnik et al. (2017) examined the effect of resveratrol on the inhibition of biofilm formation (measured as adhered biomass) by *C. jejuni* and the culturability and viability of the adhered cells post treatment with resveratrol. Sub-inhibitory concentration (12.5–200 μg/mL) of resveratrol resulted in 40% inhibition of *C. jejuni* biofilm formation and 20–30% inhibition was achieved at other lower concentrations tested. When tested at MIC, 2x MIC and 5x MIC (MIC = 313 μg/mL), resveratrol resulted in even higher percentage inhibition in biomass formation, and reduced culturability and viability of the adhered cells by 50–60%.

Resveratrol was also shown to exhibit antibiofilm activity against *Vibrio* bacteria. Augustine et al. (2014) reported concentration dependent inhibition of percentage biofilm formation in *Vibrio cholerae* O1 MCVO9; at 15–30 μg/mL, resveratrol inhibited biofilm formation by 64–85%. Reduction of cells adherent to the coverslip was visualized using confocal laser microscopy whereby the thickness of the biofilm formed was significantly different between resveratrol treated cells and control. Meanwhile, Kim et al. (2015) reported no significant reduction in percentage biofilm formation in *Vibrio cholera* O1 El Tor serotype Inada strain N16961 after treated with 10 μg/mL of resveratrol. Perhaps, a higher concentration of resveratrol is needed to inhibit the biofilm formation by *V*. cholera O1 El Tor serotype as shown by the earlier study with positive inhibitory effect by resveratrol at 15–30μg/mL against *V. cholerae* O1 MCVO9 (Augustine et al., 2014). Besides the selective antibiofilm activity demonstrated by resveratrol against *V. cholerae*, it was demonstrated that resveratrol inhibited cholera toxin induced cAMP accumulation in Vero cells through the suppression of cholera toxin internalization (Morinaga et al., 2010). Collectively, the evidence suggests that resveratrol has the potential to be developed into an anti-Vibrio agent that provides protective effects against cholera infection.

Although the existing evidence indicates that resveratrol exhibits antibiofilm properties against various foodborne pathogens, substantial efforts using reliable quantification methods are still needed in order to show that resveratrol is truly an effective antibiofilm agent. Clearly, the current evidence warrants further work to elucidate the exact mechanism of action of resveratrol in the disruption or inhibition of foodborne pathogen biofilms. Also, these antibiofilm properties of resveratrol hold promise for the development of a molecule that may be used either on its own or as an adjuvant to existing suppression or eradication strategies against foodborne pathogens.

## Application and efficacy of resveratrol in the food system

While the laboratory results are very encouraging, it should be borne in mind that there are potential limitations to the degree to which the study findings may be applied in real world situations. Food and food products are rich in complex nutrients, which may support growth of bacteria even better than nutrient media, and it is likely a higher concentration of antimicrobial might be needed to achieve the desired results even though substantial antimicrobial activity is achieved in *in-vitro* studies (Shelef, 1984; Gill et al., 2002). Intrinsic properties such as fat, protein, water content, antioxidant, pH, food additive and extrinsic properties such as storage condition, temperature, and microorganism characteristics can also potentially diminish the efficacy of the antibacterial agent (Tassou et al., 1995). Furthermore, the possibility of nutritional and organoleptic losses should also be noted when using an antibacterial agent to improve microbial quality and safety of food. For example, in the case of essential oils, despite their greater antimicrobial potential (Burt, 2004; Lai et al., 2017) than resveratrol, their application in food preservation is still limited by their intense aroma and toxicity issues.

For resveratrol, even though promising results were obtained from the *in vitro* experiments including the agar and broth systems, applied studies are also mandatory to confirm its effectiveness in food protection against foodborne pathogens. Surendran Nair et al. (2016) examined the enhancement of thermal inactivation of *E. coli* O157:H7 inoculated on beef patties in the presence of resveratrol. They reported no decline in *E. coli* count irrespective of treatment throughout the refrigerated storage (4°C for 5 days) in uncooked patties. In patties that are stored at 4°C for 5 days and cooked to an internal temperature of 65°C, significant reduction (>3 log CFU/g) of *E. coli* was observed for day 1, 3, and 5 at 0.2% w/w resveratrol in comparison to control whereas at 0.1%w/w, significant reduction was observed at day 3 and 5. A synergistic effect was observed when either concentration of resveratrol was combined with chitosan (1% w/w), which significantly reduced count, while chitosan + resveratrol 0.2% w/w completely diminished *E. coli* count in day 3 and 5 (Surendran Nair et al., 2016). They proposed the synergistic effect observed was because chitosan, being an emulsifier, can solubilise resveratrol, thereby improving the distribution of resveratrol in meat (Klinkesorn, 2013). Enhancement of thermal activation in the presence of resveratrol suggested that heat caused damage to cell membrane integrity, thereby allowing better penetration of resveratrol into cells. Similarly, resveratrol was shown to cause oxidative damage to cell membrane, which may help to enhance the killing when compared to heat alone (Subramanian et al., 2014).

Ferreira and Domingues (2016) demonstrated the antibacterial effect of resveratrol (200–400 μg/mL) against *L. monocytogenes* and *L. innocua* in four food models: UHT treated skim milk, UHT treated whole milk, chicken juice and lettuce leaf. They reported resveratrol significantly reduced bacterial count for both strains in chicken juice and lettuce leaf model. The effect was less pronounced for *L. monocytogenes* in UHT-treated skim and whole milk, with no significant reduction of bacteria in the whole milk. They proposed the observed difference was due to higher fat content in whole milk which protected the bacteria from inhibition by resveratrol. Due to the hydrophobic nature of resveratrol, it might preferentially dissolve in the lipid phase of milk, thus decreasing the amount of active substance available to act on bacteria (Mejlholm and Dalgaard, 2002).

Consumption of raw vegetables also represents a common source of food borne infections, thus it is critical to control the microbial load in the fresh produce particularly from the salad supply chain, ensuring food safety and hygiene quality. Lai et al. (2017) demonstrated the potential of resveratrol to be used as a natural preservative to control the total viable microbial load in alfalfa sprout and mushroom slices. They reported 0.9 log reduction and 3.06 log reduction in microbial load in alfalfa sprout and mushroom respectively after 24 h storage at 4°C (Lai et al., 2017).

There are some concerns with reference to resveratrol's impact on the organoleptic properties of the food, as it may impart an astringent flavor and bitterness to the food. Gaudette and Pickering (2011) reported an increase in the perception of bitterness with increasing concentrations of resveratrol added to wine. To minimize the effect of resveratrol on the organoleptic properties of the food, techniques such as encapsulation of resveratrol by spray-drying was suggested by Koga et al. (2015) to reduce the perception of bitterness. They reported a higher taste detection threshold for resveratrol (313 μg/mL) encapsulated with sodium caseinate than unencapsulated resveratrol (90 μg/mL) in protein solution (Koga et al., 2015). To date, studies on the sensory evaluation of resveratrol in the food system are still limited, thus further work is needed to improve the utilization of resveratrol as a food preservatives by minimizing its taste altering effect.

## Bioavailability, safety profile, and drug-drug interactions of resveratrol

Resveratrol was reported to be extensively absorbed and metabolized but with very low bioavailability. Walle et al. (2004) reported that 70% of an oral dose of 25 mg resveratrol was absorbed but the concentration remaining unchanged in plasma was <5 ng/ml. They proposed that this was because resveratrol was metabolized in liver and intestine by sulfate and glucuronic acid conjugation and by hydrogenation of aliphatic double bond. In addition to that, inter-individual variability of resveratrol metabolism was also reported by Bode et al. (2013), where they proposed the difference seen might be attributed to variation in human gut microflora in microbial transformation of resveratrol. Thus, to effectively utilize resveratrol in foods and pharmaceuticals, many efforts have been reported to improve the bioavailability of resveratrol through various encapsulation methods.

The safety profile of resveratrol has been well documented and is safe for consumption up to 5 g per day in healthy humans. Several studies reported that gastrointestinal side effects such as diarrhea, nausea, and abdominal pain were common when administered at high doses (Brown et al., 2010; Chow et al., 2010; Howells et al., 2011; Anton et al., 2014; Sergides et al., 2016). Brown et al. (2010) reported mild gastrointestinal symptoms when resveratrol was administered at a doses of 2.5 g/day while Sergides et al. (2016) reported resveratrol administered 500 mg/day was well tolerated in human. Similarly, Howells et al. (2011) reported mild gastrointestinal symptoms as the primary side effect experienced by patients taking resveratrol 5 g/day, with other less common side effects such as chills, rash, skin irritation and vascular flushing also reported. Anton et al. (2014) reported short-term resveratrol administration (90 days) at 300 mg/day and 1 g/day was well tolerated in overweight elderly and did not significantly altered blood chemistries. Chow et al. (2010) also reported high tolerability of resveratrol administered at 1 g/day as most side effects were mild and transient.

The potential drug-resveratrol interaction is also an important aspect requiring considerable attention. Co-administration of resveratrol together with other drugs may potentially result in drug-resveratrol interactions by changing the pharmacokinetics of the drug or resveratrol. Resveratrol inhibits several key CYP enzymes such as CYP3A4, 1A2, 2C9, 2D6, and 2E1. Thus, high intake of resveratrol may theoretically reduce the metabolism of drugs by CYP enzymes and increase their bioavailability and potential toxicity. In a clinical trial study by Chow et al. (2010), they reported 1.33-fold increase in AUC for buspirone following co-administration of resveratrol at 1 g/day for 4 weeks due to inhibition of CYP3A4. Similarly, Choi et al. (2009) reported 2.2 and 2.3-fold increase in C_max_ and AUC of nicardipine respectively in male rats following co-administration of resveratrol at 10 mg/kg body weight. Potential systemic drug-resveratrol interaction should be considered at high doses at > 1 g/day. However, the risk of interaction is minimal at low milligram doses as resveratrol has a very low bioavailability and is rapidly metabolized by conjugation *in vivo*. Therefore, the systemic concentration of free resveratrol is far below the conjugates. In addition, there have been no reports so far of inhibition of CYPs by resveratrol conjugates, and high plasma protein binding of resveratrol also reduces the risk of potential interactions (Detampel et al., 2012).

## Commercialization and future directions

At the time of writing, more than 5000 patents were found from searching “Scopus” using search terms “resveratrol” AND “antibacterial” OR “antimicrobial” limit to title/abstract/keywords. Some of the patented products containing resveratrol included antibacterial foot spray and hand cleanser (Yoo and Kim, 2013; Zhu et al., 2016), botanical fungicide (Li et al., 2015), skin care products (He et al., 2017; Lei et al., 2017), food supplements (Sardi and Solomonson, 2005; Pall, 2014; Arroyo Paz, 2017) and antioxidant eye drops (Kador, 2016). Clearly, in the future, more research is required to unravel the potential of resveratrol to be developed into biopharmaceutical products. As the studies currently focus only on *in-vitro* determination of biological activity of resveratrol, there is an urgent need for studies on the efficacy of resveratrol in *in vivo* animal models associated with foodborne infections. In addition, to obtain the full potential of resveratrol, further evaluation of the mechanism of action of resveratrol will be needed to strengthen its practical applications and significance in future research. Further investigations could be extended to interactions between resveratrol and the food ingredients or food additives as well as its effect on the organoleptic properties of the product. Studies of the stability of resveratrol under various storage conditions and processing conditions are also needed to confirm its applicability in the food industry.

## Conclusion

Food contamination by pathogenic foodborne bacteria is a major health risk to the public and the situation is being further exacerbated by the emergence of antibiotic resistant strains which are promoted by the ever-increasing usage of antibiotics. Natural antimicrobials seem to hold promise as an alternative to antibiotics and can be used to decrease the spread of resistant strains. Resveratrol exhibits promising antibacterial activities against important foodborne bacteria including *S. aureus, L. monocytogenes, C. jejuni, E. coli*, and *V. cholerae*, possibly mediated through DNA cleavage, membrane damage, reduced cellular metabolic activity and inhibition of cell division. The antibiofilm and antivirulence activities of resveratrol can be exploited as stand-alone alternative therapy or as coadjuvants to current antibiotic therapy against foodborne pathogens. Furthermore, resveratrol also proved to be effective in inhibiting several foodborne pathogens in different types of food matrix, which supports its potential use as an alternative food additive or preservative to ensure high quality food products which are safe to be consumed. Based on the current toxicological data, resveratrol was shown have low toxicity even when taken at high doses with mild gastrointestinal disturbances being the major side effect reported. Overall, this review highlights the potential application of resveratrol as an antimicrobial biopharmaceutical product as well as to have significant applications in the food industry as a food preservative.

## Authors contributions

The writing was performed by DM, LT-HT, PP, L-HL, and B-HG. While WY, LH-C, TK, K-GC, LM, L-HL, and B-HG provided vital guidance and insight to the work. The project was conceptualized by L-HL and B-HG.

### Conflict of interest statement

The authors declare that the research was conducted in the absence of any commercial or financial relationships that could be construed as a potential conflict of interest.
